# Effect of Cement Types and Superabsorbent Polymers on the Properties of Sustainable Ultra-High-Performance Paste

**DOI:** 10.3390/ma14061497

**Published:** 2021-03-18

**Authors:** Mei-Yu Xuan, Yi-Sheng Wang, Xiao-Yong Wang, Han-Seung Lee, Seung-Jun Kwon

**Affiliations:** 1 Department of Architectural Engineering, Kangwon National University, Chuncheon-si 24341, Korea; xuanmeiyu@kangwon.ac.kr; 2 Department of Integrated Energy and Infra System, Kangwon National University, Chuncheon-si 24341, Korea; wangyisheng@kangwon.ac.kr; 3 Department of Architectural Engineering, Hanyang University, Ansan-si 15588, Korea; ercleehs@hanyang.ac.kr; 4 Department of Civil and Environmental Engineering, Hannam University, Daejeon-si 34430, Korea; jjuni98@hannam.ac.kr

**Keywords:** superabsorbent polymer, belite-rich Portland cement, sustainable ultra-high-performance paste, internal curing, autogenous shrinkage

## Abstract

This study focuses on the effects of superabsorbent polymers (SAP) and belite-rich Portland cement (BPC) on the compressive strength, autogenous shrinkage (AS), and micro- and macroscopic performance of sustainable, ultra-high-performance paste (SUHPP). Several experimental studies were conducted, including compressive strength, AS, isothermal calorimetry, X-ray diffraction (XRD), thermogravimetric analysis (TGA), attenuated total reflectance (ATR)–Fourier-transform infrared spectroscopy (FTIR), ultra-sonic pulse velocity (UPV), and electrical resistivity. The following conclusions can be made based on the experimental results: (1) a small amount of SAP has a strength promotion effect during the first 3 days, while BPC can significantly improve the strength over the following 28 days. (2) SAP slows down the internal relative humidity reduction and effectively reduces the development of AS. BPC specimens show a lower AS than other specimens. The AS shows a linear relationship with the internal relative humidity. (3) Specimens with SAP possess higher cumulative hydration heat than control specimens. The slow hydration rate in the BPC effectively reduces the exothermic heat. (4) With the increase in SAP, the calcium hydroxide (CH) and combined water content increases, and SAP thus improves the effect on cement hydration. The contents of CH and combined water in BPC specimens are lower than those in the ordinary Portland cement (OPC) specimen. (5) All samples display rapid hydration of the cement in the first 3 days, with a high rate of UPV development. Strength is an exponential function of UPVs. (6) The electrical resistivity is reduced due to the increase in porosity caused by the release of water from SAP. From 3 to 28 days, BPC specimens show a greater increment in electrical resistivity than other specimens.

## 1. Introduction

Ultra-high-performance concrete (UHPC) has a high cementitious material content, low water–cement ratio (w/c), dense microstructure, and very low porosity [1]. Therefore, it has superior mechanical properties and durability performance. To achieve a high-strength and dense structure in UHPC, the mechanical properties and rheology can be improved by adding microfillers [2]. Usually, silica fume is added as a typical reactive powder for UHPC, but this causes autogenous shrinkage (AS) to be high [3]. In addition, limestone fillers and blast-furnace slag are also used to increase UHPC sustainability. The addition of limestone and slag can increase cement’s degree of hydration, improve mechanical strength, and lower CO_2_ emissions [3,4,5]. In addition, as a result of the extremely low w/c of UHPC, the final hydration value can be less than 50% [6]. AS and autogenous cracking limit the application of UHPC in practical engineering [7].

To effectively promote cement hydration and reduce AS, the internal relative humidity of UHPC can be increased by providing it with additional water [8]. However, the extremely low porosity (very low permeability) of UHPC makes it impossible for water from outside to enter [6]. UHPC was found to be more suitable than the internal curing method for maintenance [9]. Internal curing is the process of mixing and dispersing materials with high water absorption capacity into concrete and gradually releasing water to the surrounding area during the cement’s hydration process [10]. Common internal curing methods are lightweight aggregates (LWA) [11] and SAP [9]. The water absorption ability of SAP is much higher than LWA; consequently, the internal curing efficiency of SAP is higher [12,13]. Moreover, SAP is more conducive to controlling the distribution, shape, and size of defects and pores [14]. For the internal curing of UHPC, SAP can effectively maintain internal relative humidity and reduce AS [15,16,17].

SAP materials have hydrophilic networks that can absorb large amounts of water (other solutions) without being dissolved [18]. Further, SAP absorbs water and swells to form a hydrogel, providing additional water during the concrete hardening process [19]. Liu et al. [20] indicated that SAP, as an internal curing agent, could delay the appearance of cracks and reduce the early AS of UHPC. In composite cement systems with fly ash or slag, the water absorbed/released by SAP has an effective shrinkage reduction [21]. Justs et al. [6] explored the internal curing effect of SAP on UHPC, which reduced shrinkage by around 75% when 2% SAP was added. When SAP is used as an internal curing agent, it can promote hydration to improve the microstructure, but the formation of pores during the release of water also increases the porosity [22] and affects the mechanical properties [7]. Song et al. [23] indicated that SAP mitigated the internal relative humidity drop and was effective in reducing AS, but the strength of the samples was also reduced. In mortars with a w/c of 0.55, the addition of SAP reduces the early strength, but the effect gradually decreases at a later stage [24]. From previous studies, it can be concluded that SAP as an internal curing agent has excellent performance in reducing AS, but with a corresponding reduction in strength.

Although many studies have been conducted to examine SAP internal curing of UHPC, previous studies show some weak points. (1) The binder used in UHPC in previous studies mainly consists of cement and silica fume. The study of sustainable, ultra-high-performance paste (SUHPP) with other SCMs and fillers, such as limestone and slag, was insufficient. Moreover, the internal curing of SUHPP was rarely studied. (2) The cement used in UHPC in previous studies mainly consisted of type I Portland cement. Other types of cement, such as BPC, were seldom used. Compared with OPC, the CO_2_ emission of BPC is around 10% lower [25]. Moreover, BPC can reduce hydration heat, which is helpful for reducing thermal cracking. Hence, BPC is a sustainable material compared with OPC. In addition, compared with OPC, the rate of hydration of BPC is much slower, which may be helpful for reducing AS in UHPC [26]. In addition, UHPC has a high binder content and experiences a high temperature rise. The utilization of BPC can lower the temperature rise of UHPC. Furthermore, the hydration of C_2_S in BPC can enhance the late-age strength of UHPC. Due to these advantages of BPC, concrete factories were eager to know whether BPC was suitable for producing UHPC. (3) Previous studies mainly focus on the AS and strength of SAP-blended UHPC. Studies on other aspects have also been insufficient, i.e., studies regarding internal relative humidity and temperature of hardening specimens, strength monitoring with ultrasonic pulse velocity (UPV), and electrical resistivity development.

Therefore, this study focuses on the effect of adding SAP and replacing BPC in SUHPP with silica fume, limestone, and slag. Several experimental studies were conducted, including compressive strength, AS coupled with internal relative humidity and temperature, isothermal calorimetry, X-ray diffraction (XRD), thermogravimetric analysis (TGA), attenuated total reflectance (ATR)–Fourier-transform infrared spectroscopy (FTIR), UPV, and electrical resistivity.

The innovation points of this study can be summarized as follows: First, silica fume, limestone, and slag were used to produce SUHPP. The internal curing effect of SAP on SUHPP was investigated. Second, we clarified the effects of the type of cement on the differences in the performance of SUHPP. Finally, a systematical experimental investigation into hydration, AS, strength, and durability was performed.

The aims of the research questions in our research are (1) to find feasible methods for reducing the AS of SUHPP and to clarify the mechanism of AS in SUHPP; (2) to conduct detailed and various experimental studies on SUHPP containing SAP and BPC and to explore the relations among the various results; and (3) to discuss the strong points and weak points of paste containing SAP or BPC and to determine the expected practical applications of the tested specimens.

## 2. Materials and Experimental Methods

### 2.1. Material and Ratio Design

**Figure 1:**
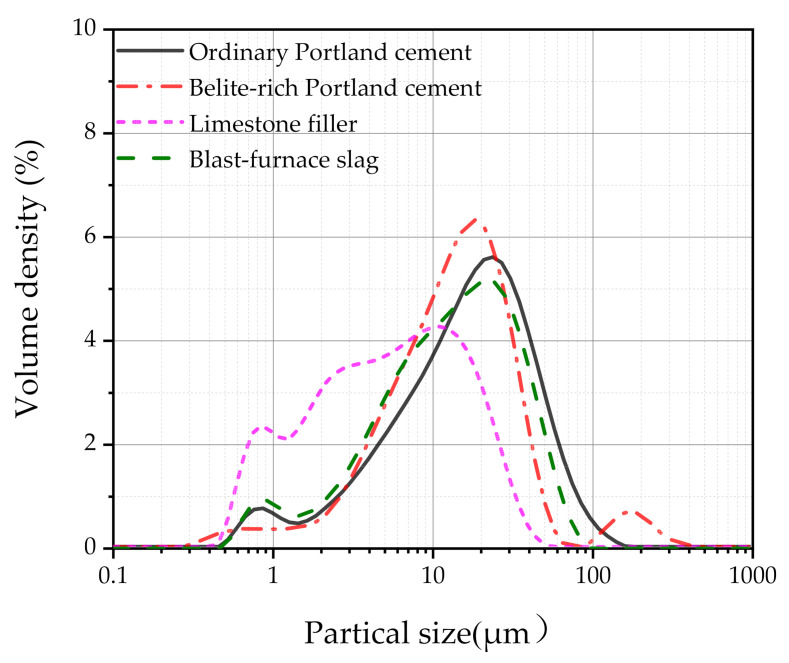
Particle size distribution of each component.

**Figure 2:**
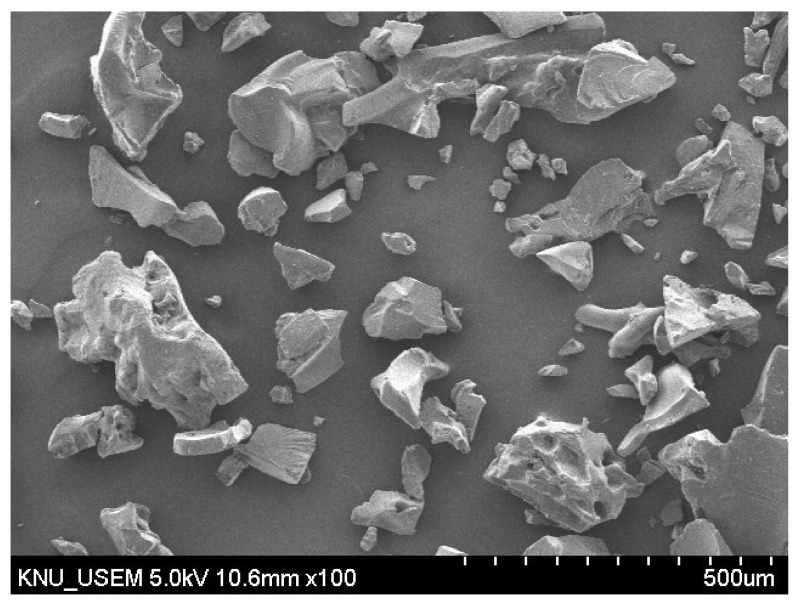
Scanning electron microscope (SEM) images of dried superabsorbent polymer (SAP) particles (magnification scale is 100).

In this study, SUHPP was prepared with a fixed water–binder ratio of 0.2. The chemical composition of OPC, BPC, silica fume, limestone, and slag used in mixtures is listed in Table 1. Based on the chemical composition listed in Table 1 and calculated by the Bogue formula, the mineralogical composition of the clinker is shown in Table 2. The particle size distribution of each component is shown in Figure 1. According to Figure 1, the average particle sizes of OPC, BPC, limestone, and slag were 16.4, 14.5, 5.21, and 12.7 μm, respectively. In addition, the water absorption of SAP was measured using the “tea bag” method [27]. In water, the swelling capacity of SAP was equal to 295.5 g/g_SAP_ after 10 min and 291.5 g/g_SAP_ after 12 h. The swelling capacity of SAP in cement paste was much lower than when in water [12]. In cement paste, the swelling capacity of SAP was approximately 10.4 g/g_SAP_. A scanning electron microscope (SEM) (S-4800, Hitachi, Tokyo, Japan) image of the SAP is shown in Figure 2. In general, SAP particles have an irregular shape.

Regarding the quaternary mixture, the weight ratio of silica fume, limestone, and slag was 1:2:2. As shown in Table 3, the effect of SAP content was studied. We added 0.25% and 0.5% (% by mass of binder) SAP content, respectively. In addition, the effect of the cement type was studied using OPC–0SAP and BPC–0SAP. In order to obtain proper workability, 1.2% (% by mass of binder) by weight of superplasticizer (SP) was added.

### 2.2. Mixture Preparation Method

**Figure 3:**
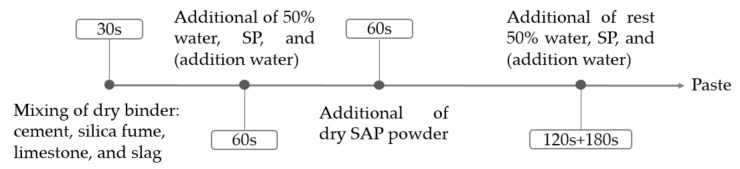
Mixing procedure for composite mixture paste.

Figure 3 shows the specific steps of the mixing procedure of SUHPP. Step 1: dry mix all the binding materials for 30 s. Step 2: add 50% of water, SP, and/or additional water, mixing for 60 s. Step 3: add the dry SAP, mixing for 60 s. Considering that the presoaked SAP turns into a hydrogel, it is difficult to disperse in the mixture [12]. Therefore, it is reasonable to add the dry SAP directly [12]. Step 4: add the rest of the water, SP, and/or additional water; mix slowly for 120 s; and then mix quickly for 180 s.

### 2.3. Experimental Methods

Table 4 lists the experimental methods used in this study and the range of tests performed. First, we measured the compressive strength of the sample based on ASTM C39 [28]. Second, the AS coupled with internal relative humidity and temperature measurements were performed using a bellows self-shrinkage tester (Instrument Creation Era, Beijing, China) (based on American Standard ASTM C1698-09) [29]. The isothermal calorimeter TAM Air (TA Instruments, New Castle, CO, USA) was used to measure the heat flow and cumulative hydration heat of the samples (mixing out-of-bottle). After 28 days, XRD analysis was conducted using PANalytical X’pert pro MPD diffractometers (Panalytical, Almelo, The Netherlands). Scanning measurements were performed in the 2θ range from 5° to 75° in steps of 0.02° under CuKα radiation (λ = 1.5404 Å) [30]. At 28 days, a thermal analysis system (SDT Q600, TA Instruments, Santa Clara, CA, USA) was used for TGA. We used temperatures ranging from 20 to 1050 °C at a rate of 10°/min. At 28 days, the samples were scanned using a frontier spectrometer (PerkinElmer, Waltham, MA, USA). The resolution was 0.4 cm^−1^, and each scan ranged from 2000 to 500 cm^−1^ (7–8). Moreover, at 1, 3, 7, and 28 days, the ultra-sonic pulse velocity and electrical resistivity evolution of the samples were recorded using a nondestructive digital indicator tester (Pundit Lab, Proceq Company, Schwerzenbach, Switzerland) and a four-point Wenner probe surface testing device (Proceq Company, Schwerzenbach, Switzerland).

## 3. Experimental Results

### 3.1. Compressive Strength

**Figure 4:**
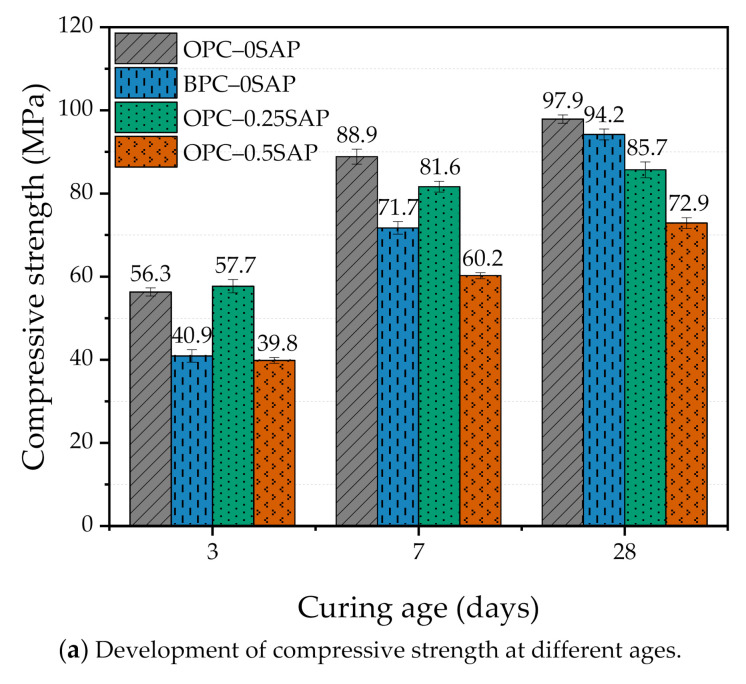

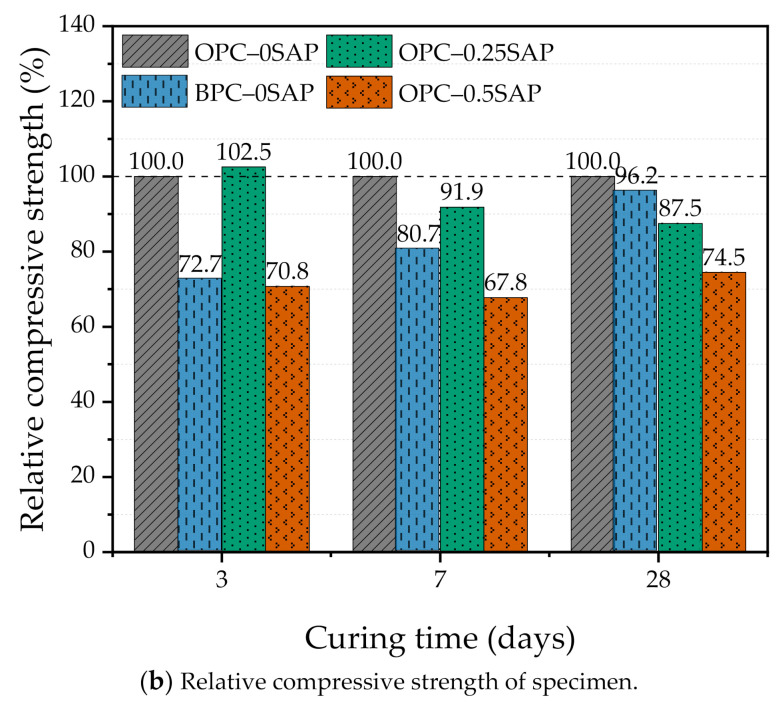
The effect of the amount of SAP added at different ages (3, 7, and 28 days) and the type of cement on the compressive strength. (**a**) Development of compressive strength at different ages; (**b**) relative compressive strength of specimen.

Figure 4 shows the effects of the amount of SAP added and the type of cement on the compressive strength development of the mixture samples at 3, 7, and 28 days. The addition of SAP to a mixture has both pros and cons. First, SAP becomes a hydrogel by absorbing water and swelling in the mixing process. In the process of resolution, larger pores are formed, resulting in lower strength [6]. Secondly, SAP releases the absorbed water to the surrounding area in the reaction process (w/c decreases), which plays the role of internal curing, increases hydration, improves the microstructure (pore densification), and promotes the development of strength [17,23]. In general, the effect of SAP on the development of strength depends on the competing effects of SAP on the increase and decrease in compressive strength. When BPC is used instead of OPC, the structure of belite (e.g., irregular structure, few cavities, and low activity) leads to a slow strength increase at an early age and a faster strength increase at a later age [31].

At 3 days of curing age, the compressive strength of OPC–0.25SAP was slightly higher than that of the control group due to the improved degree of hydration from the internal curing of water. The compressive strength of OPC–0.5SAP samples was 29.2% lower than that of the control group due to the increase in porosity caused by SAP [16]. Compared with higher content, a lower amount of SAP was observed for the early-age strength.

At 7 and 28 days of curing age, the compressive strength of the samples with SAP was lower than that of the control group. At 7 days, the compressive strengths of OPC–0.25SAP and OPC–0.5SAP were 8.1% and 32.2% lower than that of the control group, respectively. At 28 days, the compressive strengths of OPC–0.25SAP and OPC–0.5SAP were 12.5% and 25.5% lower than that of the control group, respectively. This indicates that the internal curing of SAP at a later age improves hydration, promotes the pozzolanic reaction, and effectively increases the compressive strength [7,16]. However, the SAP internal maintenance effect does not fully compensate for the strength loss caused by the voids created by the SAP water release [7]. The higher the amount added, the more obvious the reduction phenomenon.

In contrast, the effect of the replacement cement type on strength ranged between 0.25% and 0.5% of SAP. At 3 and 7 days of hydration, the strength of BPC–0SAP was only 72.7%–80.7% of OPC–0SAP. This was attributed to the slow hydration rate of C_2_S in BPC–0SAP, resulting in a slow increase in strength in the early stages [32]. Although the early compressive strength developed slowly due to the higher C_2_S and lower C_3_A in the BPC cement, the later (28 days) strength was significantly improved [33]. The compressive strength of BPC–0SAP samples at 28 days could reach 96.2% of the control group. For strength, the BPC–0SAP samples had a higher late-stage strength than the samples doped with SAP.

### 3.2. Autogenous Shrinkage (AS) Coupled with Internal Relative Humidity and Temperature

**Figure 5:**
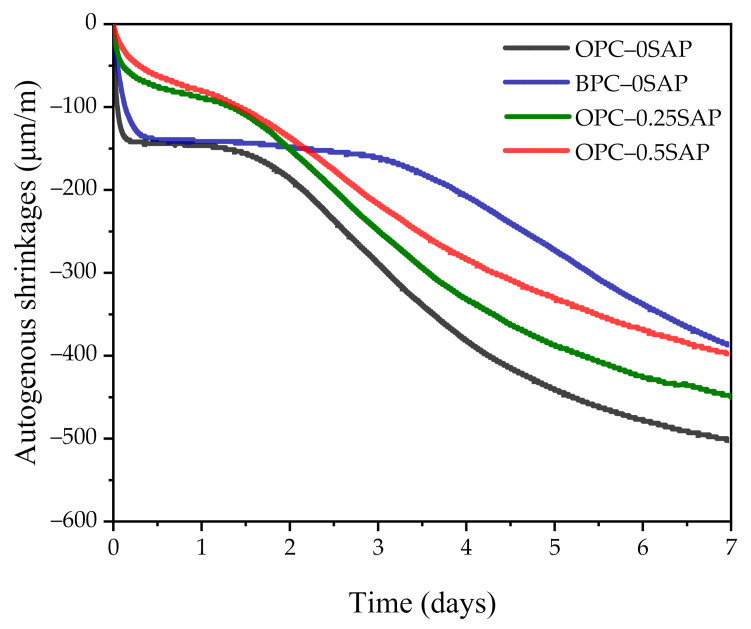
Autogenous shrinkage (AS) of sustainable ultra-high-performance paste (SUHPP) hydrated for 7 days.

As shown in Figure 5, the development of AS can be divided into the following three stages: rapid growth period at initial ages, stable period at middle ages, and continuous growth period at later ages. There are five reasons for the change in AS: (i) cement hydration consumes water and the internal relative humidity decreases, resulting in a rapid increase in AS (shrinkage factor) [12]; (ii) the densification of the internal pore structure, resulting in increased AS (shrinkage factor) [33]; (iii) the pozzolanic reaction of silica fume and slag to produce dense C-S-H, as well as to promote AS development (i.e., shrinkage factor) [34]; (iv) the production of AFm and internal temperature changes leading to expansion (expansion factor) [35]; (V) limestone stabilizes the produced ettringite and causes expansion (expansion factor) [36,37]. The final macroresponse depends on changes in the dominant expansion and contraction factors.

**Figure 6:**
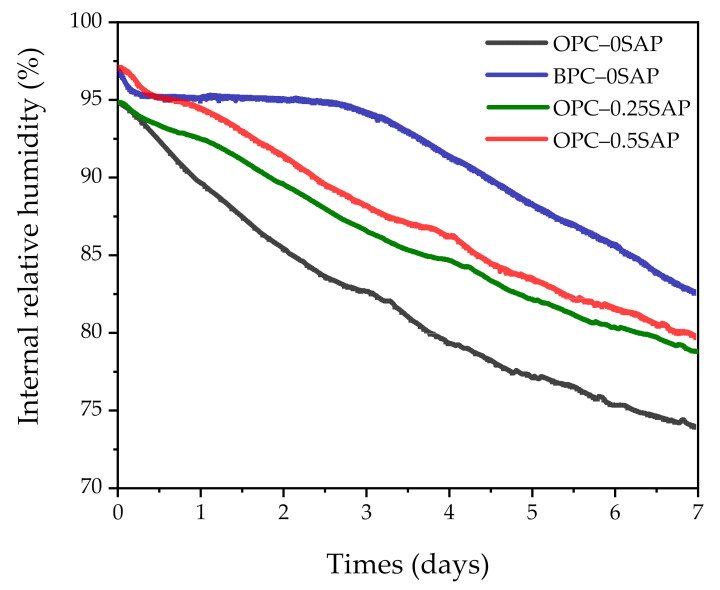
Variation in internal relative humidity with the age of curing.

At the initial ages, the AS contraction of all samples was significantly greater, while the AS of samples with SAP was significantly lower than that of the control group. This was due to the release of water from SAP during the reaction process, slowing down the decrease in internal relative humidity and effectively reducing AS development [12]. This decrease became more pronounced as the SAP content increased. It can be observed from Figure 6 that the internal relative humidity of samples containing SAP was significantly higher than that of the control at the beginning of the reaction.

In the stable period of middle ages, it was clearly observed that the OPC–0SAP and BPC–0SAP samples were stable and lasted longer than the samples with SAP. The macroperformance was more stable because the factors causing expansion and contraction offset each other.

During the continuous growth period of the late ages, the AS continued to increase as the reaction proceeded. The rate of AS development was significantly accelerated in the BPC–0SAP sample. Consistent with the strength trend, it showed a faster development trend at a later age. The AS of the BPC–0 samples at 7 days was lower than that of the samples doped with SAP. From the experimental results, it can be seen that the BPC can effectively reduce AS.

At 7 days of age, the AS values of OPC–0SAP, OPC–0.25SAP, and OPC–0.5SAP were –504.1, −447.6, and −399.2 μm/m, respectively. Compared with the control group, the reduction ratios of AS for OPC–0.25SAP and OPC–0.5SAP were 11.2% and 20.8%, respectively. Moreover, as shown in Figure 4, compared with the control group, the reduction ratios of strength for OPC–0.25SAP and OPC–0.5SAP were 8.1% and 32.2%, respectively. For the case of OPC–0.25SAP, the reduction in the AS sample was greater than the strength, while, for the case of OPC–0.5SAP, the reduction in the AS sample OPC–0.5SAP was lower than the strength. It should be noted that strength and AS are measured by different units, and they have different levels of importance. It is not wise to judge the benefit based on the comparisons between the strength loss ratio and AS reduction ratio.

At the age of 7 days, the AS values of OPC–0SAP and BPC–0SAP were −504.1 and −388.5 μm/m, respectively. Compared with the control group, the reduction ratio of AS for BPC–0SAP was 22.9%. Moreover, as shown in Figure 4, compared with the control group, the reduction ratio of strength for BPC–0SAP was 19.2%. The case of BPC–0SAP demonstrated that the reduction in AS was more obvious than for the strength.

**Figure 7:**
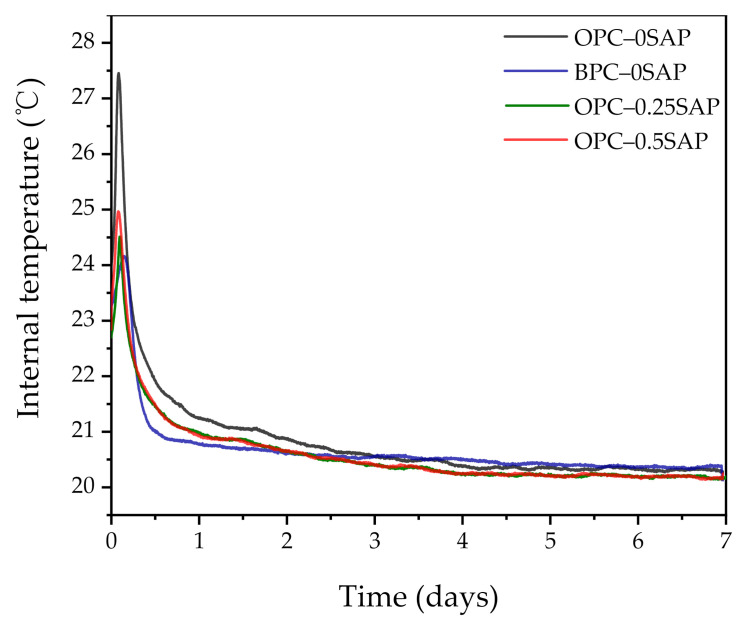
Variation in internal temperature with the age of curing.

Overall, the addition of SAP effectively reduced AS. The AS in the samples doped with BPC developed slowly and increased rapidly after the stable period. This trend is consistent with the internal relative humidity variation shown in Figure 6. The internal relative humidity of all samples gradually decreased when the curing age increased. This was due to the hydration of the cement and the decrease in water in the capillaries. However, the samples with SAP significantly delayed the rate of internal relative humidity reduction. In addition, the BPC-doped samples also retarded the reduction in internal relative humidity due to the slow reaction rate of C_2_S [38]. The low reaction rate of the BPC–0SAP sample at the early stage could be verified from the internal temperature shown in Figure 7. The internal temperature of the BPC–0SAP sample was lower compared to the other samples.

**Figure 8:**
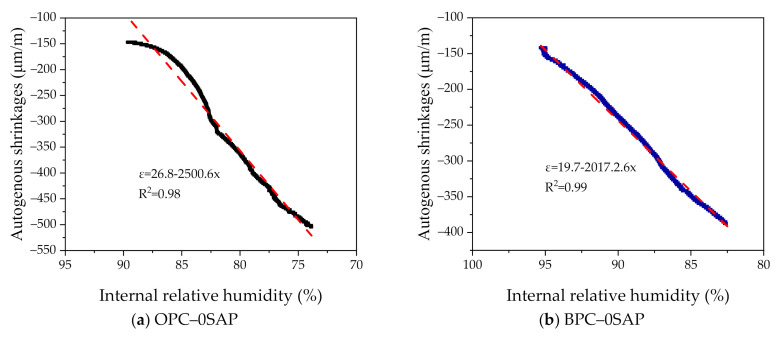

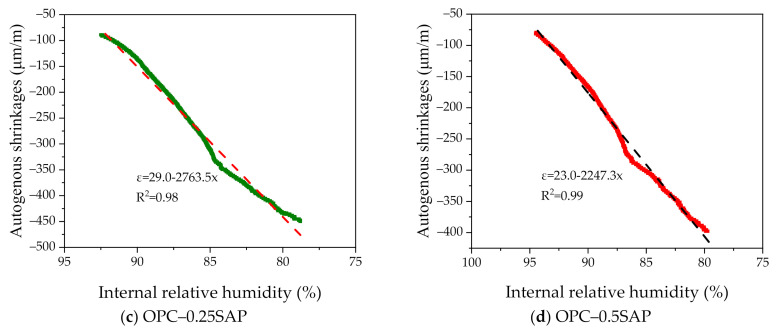
Correlation between AS and the internal relative humidity of specimen.

As shown in Figure 7, after the age of 1 day, the internal temperature was almost constant. We plotted the AS as a function of internal relative humidity from days 1 to 7. As shown in Figure 8, AS showed a linear relationship with the internal relative humidity. The coefficients of determination between AS and internal relative humidity were higher than 98%; hence, the reduction in internal relative humidity is the main reason for AS in SUHPP.

### 3.3. Isothermal Calorimetry

**Figure 9:**
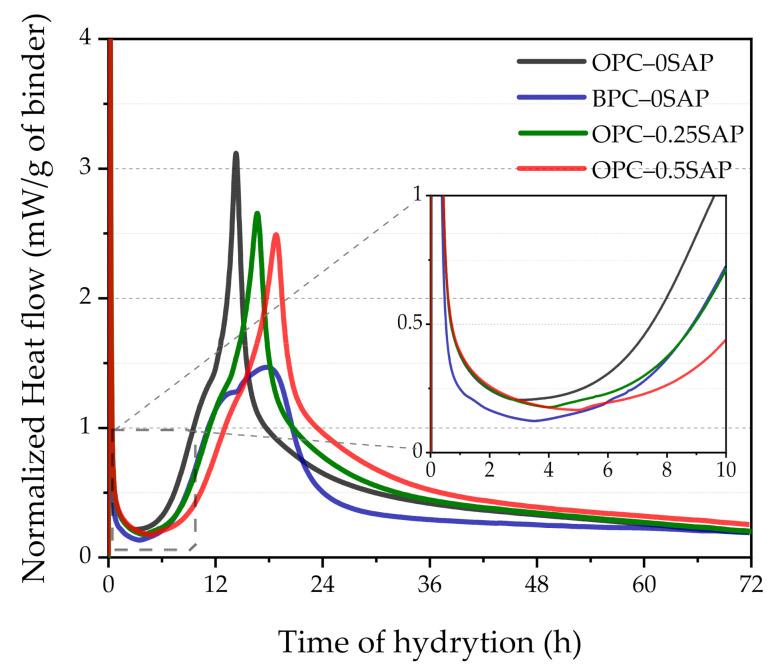
Exothermic rate of hydration of the mixed paste at 20 °C.

**Figure 10:**
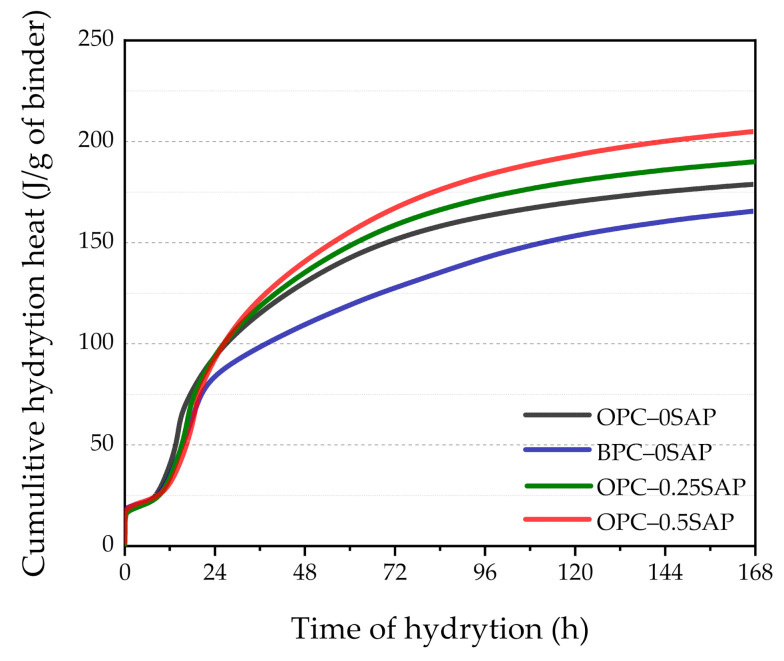
Cumulative heat change of the mixed paste during 72 h of mixing.

Figure 9 and Figure 10 show the rate of hydration heat and cumulative hydration heat change in the mixture. We found that hours 1–7 represented the dormant period, which was due to the increase in the effective w/c of samples OPC–0.25SAP and OPC–0.5SAP [39,40]. Yang et al. [41] indicated that when w/c increased, the transition from dormancy to the acceleration period was prolonged, which was consistent with the results obtained in this experiment.

The acceleration period occurred from hours 7 to 19. The peak of the main peak decreased significantly when SAP increased and became blunter. Moreover, the appearance of the main peak was delayed by 2.5–4.5 h due to the rapid resolution of SAP, which increased the effective w/c and reduced the initial reduction concentration in the pore solution, resulting in delayed cement hydration [41]. The experimental results of Justs et al. [40] indicated that within 30 h of the initial reaction, a reduced w/c shortened the dormancy period, and the main peak appeared earlier and more sharply. In addition, the BPC–0SAP sample showed a shoulder peak whose peak was higher than the peak of the main peak. The content of C_3_S in BPC was lower than OPC, resulting in a lower main peak in the BPC–0SAP sample than in the OPC–0SAP. In addition, limestone reacts with alumina in slag to form calcium alumina, thus forming a shoulder peak [42]. Shoulder peaks were also present in the other three groups of samples, but the main peaks and shoulder peaks were close to each other and did not show obvious shoulder peaks [34]. The heat flow of all samples stabilized after 36 h of hydration reaction.

As shown in Figure 10, the cumulative heat of hydration increases with the addition of SAP. The cumulative heat of hydration of both samples OPC–0.25SAP and OPC–0.5SAP was higher than that of the control after 24 h. The water entrained in SAP increased the w/c, and the higher w/c increased the degree of hydration of the sample, resulting in a higher cumulative hydration heat [43]. In contrast, the cumulative hydration heat of the BPC–0SAP samples was much lower compared to the control group. This was due to the fact that the early C_2_S hydration reaction was slower than C_3_S, releasing less hydration heat [33]. The cumulative hydration heat was released at 72 h and reduced by 15.9% compared to the control group. Further, it had much lower cumulative hydration heat than the samples mixed with SAP. The trend of cumulative hydration heat generally agreed with that of the internal temperature, as shown in Figure 7.

### 3.4. X-ray Diffraction (XRD) and Thermogravimetric Analysis (TGA)

**Figure 11:**
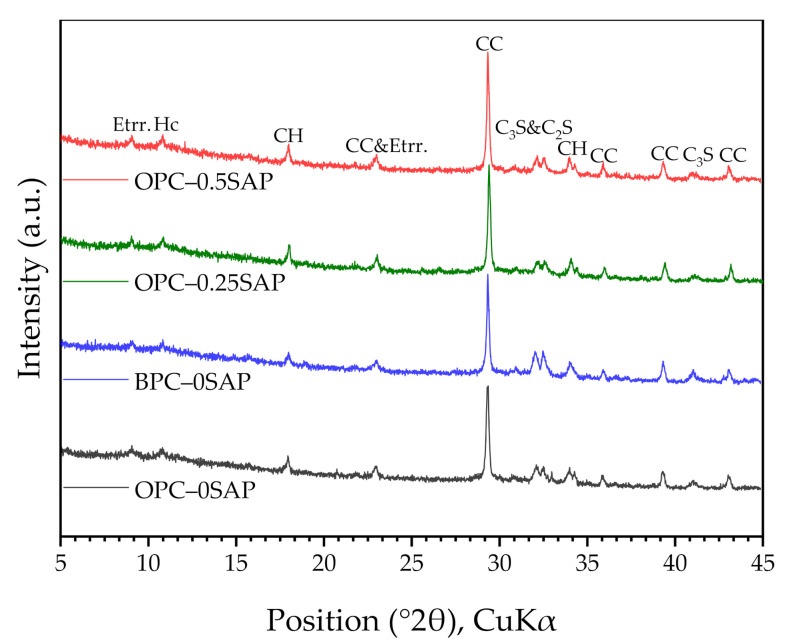
X-ray diffraction (XRD) spectra of the mixed cement paste at 28 days. Ettr.: ettringite; Hc: hemicarboaluminate; CH: calcium hydroxide; CC: calcite.

Figure 11 shows the XRD spectra of the mixed cements at 28 days of hydration. It can be observed from the plots that the peaks of Ca(OH)_2_ in OPC–0.25SAP and OPC–0.5SAP were higher than OPC–0SAP. This was due to the release of water from SAP, which promoted the hydration reaction and produced more hydration product [16]. Moreover, compared with OPC, BPC had lower reactivity, and the content of calcium hydroxide (CH) in BPC-0SAP was lower than that in OPC–0SAP. The peak of C_2_S in BPC–0SAP was higher than other specimens. In addition, the formation of hemicarbonaluminate (Hc) was due to the reaction between the alumina phase and limestone powder.

**Figure 12:**
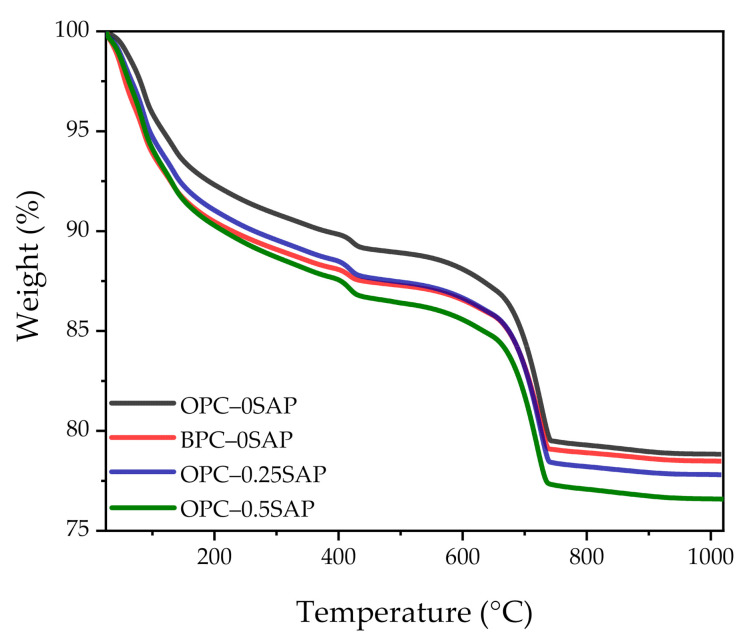
Thermogravimetric analysis (TGA) curves of all samples.

**Figure 13:**
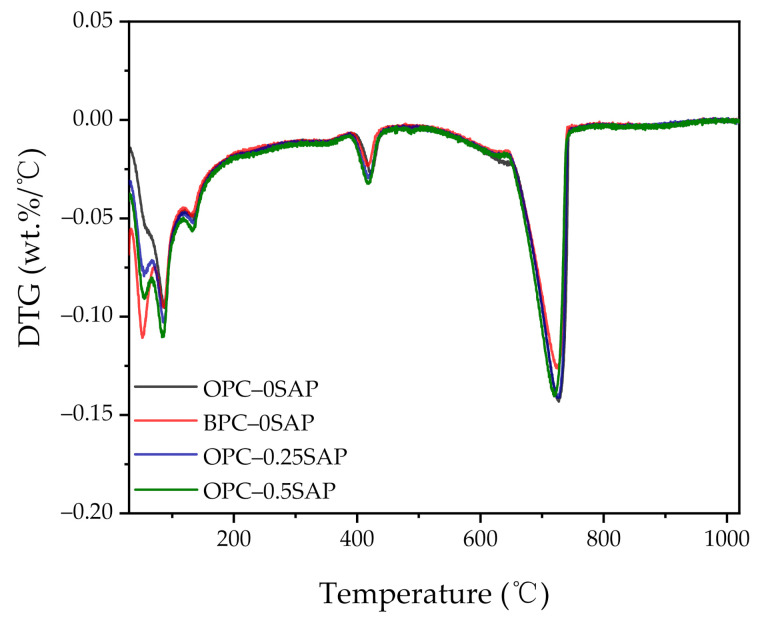
Derivative thermogravimetric analysis (DTG) curves of all samples.

In addition to the XRD spectral scan, the amount of hydration product in the samples was analyzed using thermal analysis techniques. Figure 12 and Figure 13 show the TGA and derivative thermogravimetric analysis (DTG) curves of the samples for 28 days. Weight loss can be observed in the temperature ranges of 400–450 °C and 650–750 °C, corresponding to dehydroxylation of CH and decarbonation of CaCO_3_, respectively [38,44]. The amount of CH was calculated as follows [45]:

(1) $$\mathrm{CH}=\frac{w_{400}-w_{450}}{w_{550}}\times \frac{74}{18}\times 100\%$$

where $w_{400}$ and $w_{450}$ are the masses of samples at the temperatures of 400 and 450 °C, respectively. The item 74/18 indicates the ratio of CH molar weight to water.

The amount of combined water was calculated as follows [45]:

(2) $$\mathrm{water}=\frac{w_{105}-w_{550}}{w_{550}}\times 100\%$$

where $w_{105}$ is the mass of samples at the temperature of 105 °C.

Based on the above equations, the masses of CH and combined water (W) can be determined and are shown in Table 5.

Regarding the CH content, our experimental results showed that CH_OPC-0.5SAP_ > CH_OPC-0.25SAP_ > CH_OPC–0SAP_ > CH_BPC–0SAP_. The increase in CH content with increasing SAP content indicates that the addition of SAP increased hydration. Moreover, the samples with BPC had a lower CH content compared to the control group because the reactivity of the BPC was lower than that of the OPC. The TGA analysis results showed agreement with the XRD results.

Regarding the combined water content, our experimental results show that W_OPC-0.5SAP_ > W_OPC-0.25SAP_ > W_OPC–0SAP_ > W_BPC–0SAP_. The trend of combined water was similar to that of CH. The combined water content was much less than 25%, which was the theoretical maximum of the combined water for the full hydration of 1 g cement. This was due to the low water/binder ratio and dilution effect of mineral mixtures, such as silica fume, slag, and limestone. A low water/binder ratio restricts cement-based materials from hydrating, and the addition of the mineral admixture can lower the combined water content.

### 3.5. Attenuated Total Reflectance (ATR)–Fourier-Transform Infrared Spectroscopy (FTIR)

**Figure 14:**
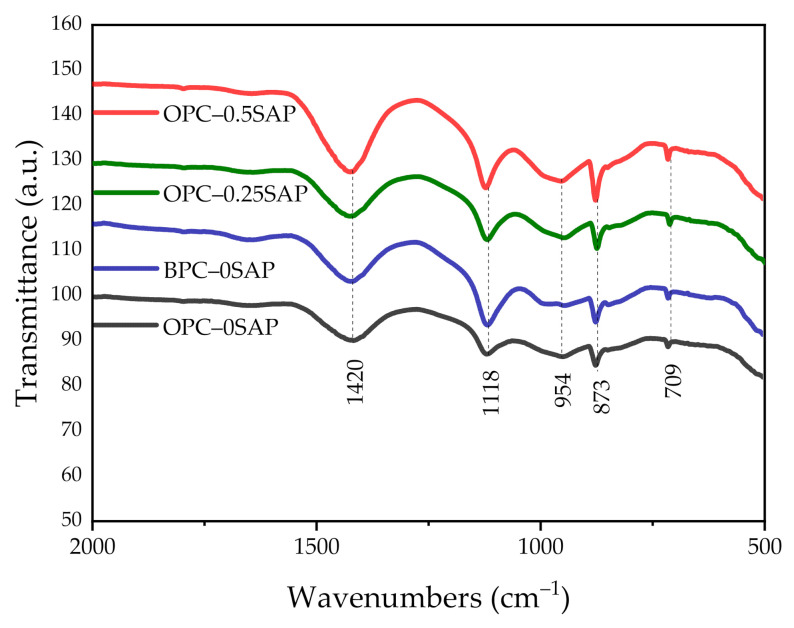
ATR–FTIR spectra of all samples for 28 days.

Figure 14 shows the ATR–FTIR spectra of the mixed samples in the range of 500–2000 cm^−1^. The 1420, 873 (out-of-plane vibration), and 709 cm^−1^ (in-plane bending) spectra in the scanned spectra correspond to CO_3_^2−^ (limestone) [46,47,48,49]. The 1118 and 954 cm^−1^ spectra represent the asymmetric stretching vibration of SO_4_^2−^ and the stretching vibration of Si-O at C-S-H, respectively [50,51]. The absorption peaks of the spectrum corresponding to the samples containing SAP at 954 cm^−1^ were significantly greater than those of the other two groups. The relative absorption peaks more obviously increased when the SAP content increased, which was attributed to the reaction of the C_3_S clinker at 800–970 cm^−1^ and the C-S-H formed by silica polymerization [46,52].

### 3.6. Ultrasonic Pulse Velocity (UPV)

**Figure 15:**
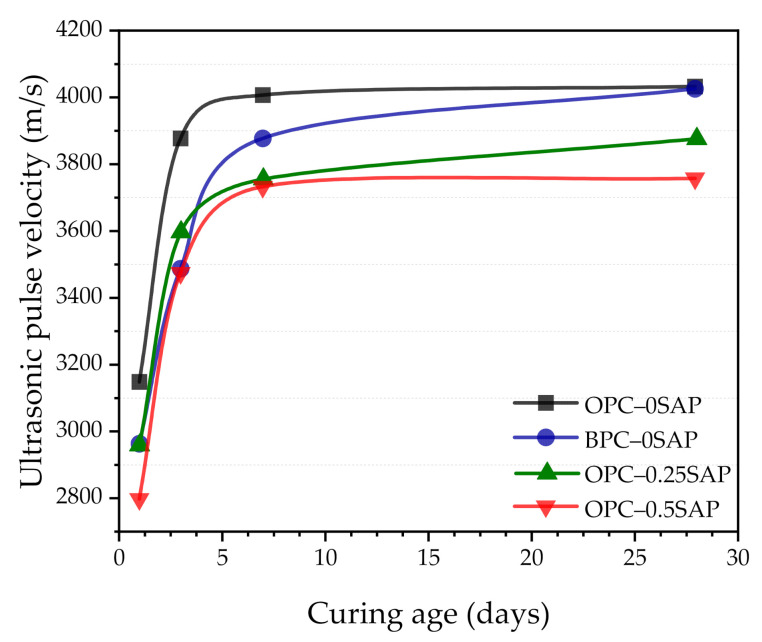
Variation in ultrasonic pulse velocity (UPV) of the mixture with the curing age.

Figure 15 shows the UPV mixture trend. The evolution of UPV was not only related to the density and elasticity of the material but also to the water content [53]. Higher SAP content makes higher effective w/c, and lower UPV [54,55]. After the dormant period, cement hydration produced a large amount of hydration product, wherein the degree of hydration increased, and the mixture had a lower porosity and a higher solid volume fraction, leading to an increase in UPV [55,56].

**Figure 16:**
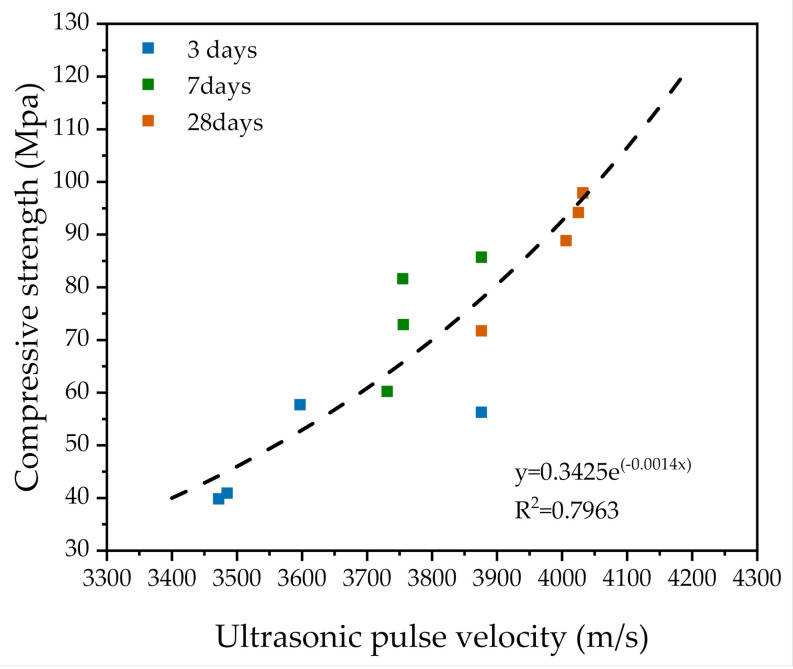
Correlation between UPV and compressive strength corresponding to 3, 7, and 28 days of hydration.

The UPV for all samples increased when curing age increased. There was fast growth at the early age and slow growth at the later age. The UPV of the OPC–0SAP sample was higher than that of the OPC–0.25SAP and OPC–0.5SAP samples. The difference gradually became larger as the SAP content increased. This was due to the fact that the higher the SAP addition and w/c, the lower the UPV. The BPC–0SAP sample grew at a lower rate than the other three sample groups (OPC–0SAP, OPC–0.25SAP, and OPC–0.5SAP) at the early age, but the growth rate significantly increased at the later age due to the high content of C_2_S contained in the BPC, which resulted in a slow initial reaction. C_2_S reacted in the late stages of hydration, increasing the degree of hydration and helping to increase the UPV at the later age [57,58]. This was in line with the trend of strength. The compressive strength of the samples at 3, 7, and 28 days regressed against the UPV, as shown in Figure 16. The strength was found to have a high exponential correlation with UPV. UPV can be a non-destructive test method for evaluating the strength development of SUHPP.

### 3.7. Electrical Resistivity

In this study, durability was evaluated based on electrical resistivity tests. For cement-based materials, as electrical resistivity increases, chloride diffusivity decreases and the service life of structures increases. Moreover, the increase in the electrical resistivity can lower the corrosion current, which is helpful for extending the service life.

**Figure 17:**
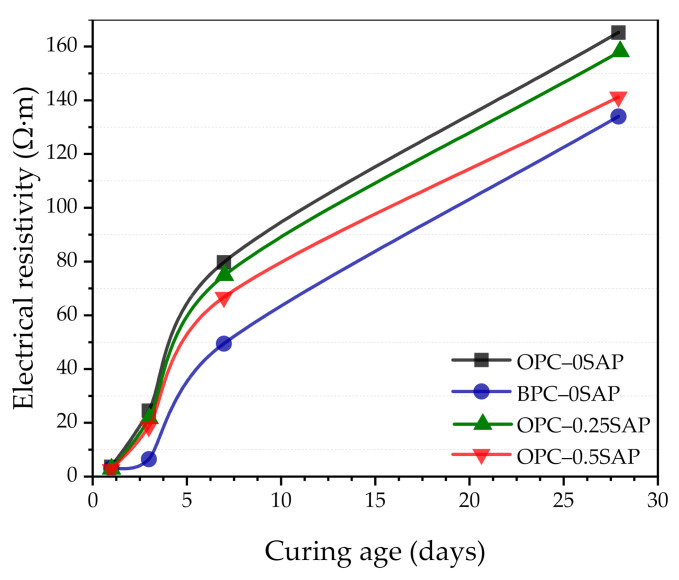
Changes in electrical resistivity of samples at different curing ages.

Figure 17 shows the evolution of the samples’ electrical resistivity with the curing age. Electrical resistivity was mainly influenced by porosity and pore structure (microstructural characteristics) [59]. SAP was added to the mixture to act as an internal curing agent and to promote hydration. However, SAP forms large pores during the resolution process, resulting in increased porosity. The negative effect of porosity on electrical resistivity far exceeds the promoting effect of internal curing [51]. The C_2_S of BPC was hydrated at a later age and gradually refined the pore structure, resulting in a gradual increase in electrical resistivity [33].

The electrical resistivity of the samples OPC–0.25SAP, OPC–0.5SAP, and BPC–0SAP was lower than that of the control samples. The higher the admixture of SAP, the lower the electrical resistivity. This is attributed to the increased w/c of the samples with SAP and the increased porosity due to SAP resolution. In addition, the electrical resistivity of the BPC–0SAP sample was significantly lower than that of the other samples. However, a gradual increase in the growth rate was clearly observed with the increase in the curing age. This was due to the late C_2_S hydration, which gradually improved the microstructure. Wang et al. [60] also showed that cements containing higher C_2_S have a much denser pore structure at a later age, which can improve the properties of the mixture. A gradual acceleration of the resistivity growth rate of BPC–0SAP can be clearly observed in later stages. The densification of the pore structure also promoted the strength development (shown in Figure 4).

## 4. Discussion

The discussion items consist of (1) the comparison of SAP with classic fibers, (2) comparison of BPC with OPC, (3) comparison of the AS reduction mechanisms of SAP and BPC, (4) the expected practical application of SAP and BPC, and (5) future studies.

First, regarding AS reduction, SAP shows superior performance to classic polyester fibers. The mechanisms of AS reduction for SAP and classic polyester fibers are different. SAP can replenish water through internal curing, and classic polyester fibers can serve as internal restraints of AS [26]. Moreover, SAP affects the technological processes of concrete mixing. The mixing procedure of SAP-blended concrete is slightly more complex than that of traditional concrete, because the presoaked SAP turns into a hydrogel and is difficult to disperse in the mixture [12]. As shown in the mixing procedure (Figure 3), Step 2 involves adding 50% of water, SP, and/or additional water and mixing for 60 s, and Step 3 involves adding dry SAP powder into the mixer. In addition, compared with classic polyester fibers, the cost of SAP is much higher. However, considering the superior performance of SAP regarding AS reduction, SAP is a promising material for AS reduction in SUHPP.

Second, BPC specimens present lower AS than OPC specimens. Furthermore, BPC specimens have a lower hydration heat, a lower CH and combined water content, less electrical resistivity, and a similar late-age strength and UPV to OPC specimens. Moreover, because BPC concrete has a higher late-age strength than OPC concrete, BPC concrete has a stronger abrasion resistance [61]. In addition, compared with OPC, the cost of BPC is slightly higher. However, considering the advantages of BPC, such as low AS, low hydration heat, low CO_2_ emission, and high late-age strength, we believe that BPC is a promising material for the production of SUHPP.

Third, the mechanisms of AS reduction in SAP and BPC are different. SAP can be used for internal curing, and BPC can control the rate of hydration [26]. The AS reduction level of SAP can be designed through SAP content, absorption ability, and particle size [26]. The AS reduction level of BPC mainly depends on the reactivity of BPC. Moreover, the addition of SAP impairs late-age strength, while BPC concrete has a similar strength to OPC concrete.

Fourth, based on the experimental results of this study, SAP can be used as a component of high-strength concrete which has a low water/binder ratio [37]. SAP can lower the AS of hardening high-strength concrete and mitigate cracks due to AS. Moreover, BPC can be used as a binder of high-strength concrete and mass concrete because BPC can lower the AS of high-strength concrete and hydration heat [61]. BPC is helpful for the mitigation of early-age AS cracks and thermal cracks.

Fifth, this study focuses only on the basic properties of SUHPP. In future studies, more investigations should be carried out to evaluate other highly important material properties—for example, tensile strength, modulus of elasticity, and fracture energy.

## 5. Conclusions

This study presents a systematical experimental investigation of the effects of SAP and cement type on SUHPP performance. Four group specimens were produced: OPC–0SAP, OPC–0.25SAP, OPC–0.5SAP, and BPC–0SAP. The SAP contents of OPC–0SAP, OPC–0.25SAP, and OPC–0.5SAP were 0%, 0.25%, and 0.5%, respectively. The cement types used in OPC–0SAP and BPC–0SAP specimens were ordinary Portland cement and belite-rich Portland cement, respectively. Experimental items consisted of compressive strength, AS, isothermal calorimetry, XRD, TGA, ATR–FTIR, UPV, and electrical resistivity. The following conclusions were obtained:

(i) At the early age of 3 days, the strength of OPC–0.25SAP samples was 2.5% higher than that of the control specimen. At the later age of 28 days, the compressive strengths of OPC–0.25SAP and OPC–0.5SAP were 12.5% and 25.5% lower than that of the control specimen, OPC–0SAP. This was due to the water release cavities created by SAP. In contrast, the strength of BPC–0SAP developed slowly at an early age, while the strength was similar to that of the control specimen, OPC–0SAP, at the later age of 28 days.
(ii) For the case of OPC–0.25SAP and BPC–0SAP, compared to the control group, the reduction in the AS sample was greater than the strength. However, for the OPC–0.5SAP sample, AS reduction was lower than the strength. Moreover, AS showed a linear relationship with the internal relative humidity. The coefficients of determination between AS and the internal relative humidity were higher than 98%. The reduction in internal relative humidity was the main reason for AS in the SUHPP.
(iii) In terms of hydration heat, the additional water entrained by SAP increased the effective w/c and reduced the initial concentration of the pore solution, resulting in a delayed and blunter main peak of 2.5–4.5 h. The higher w/c increased the degree of hydration, and the cumulative hydration heat at 72 h was significantly higher than that of the control group. The mixture doped with BPC released 15.9% lower cumulative hydration heat at 72 h compared to the control group due to the slow rate of C_2_S development responses.
(iv) TGA showed the following sequence of CH content: CH_OPC-0.5SAP_ > CH_OPC-0.25SAP_ > CH_OPC–0SAP_ > CH_BPC–0SAP_. When the SAP content increased, the CH content also increased, indicating that the addition of SAP increased hydration. The samples with BPC had a lower CH content compared to the control group. The analysis results of CH from TGA showed agreement with those of the XRD. Moreover, TGA analysis showed that the trend of combined water was similar to CH. The combined water content was much lower than the theoretical maximum of combined water for the full hydration of 1 g cement. In addition, the ATR–FTIR results showed that the Si-O stretching vibration of C-S-H was enhanced when the SAP content increased.
(v) In terms of UPV, the samples showed faster growth in 3 days because they were able to reach 86.3%–96.1%. For all specimens, there was an exponential correlation between compressive strength and UPV. UPV is a non-destructive test method for evaluating the strength development of SUHPP.
(vi) In terms of electrical resistivity, the OPC–0.25SAP and OPC–0.5SAP samples showed lower electrical resistivity than the control group due to the increased porosity from SAP. The BPC–0SAP sample showed lower resistivity in the early stage. However, from 3 to 28 days, the electrical resistivity of BPC–0SAP showed a higher increment than other specimens.

In summary, as the content of SAP increases, the AS, strength, UPV, and electrical resistivity decrease, and the hydration heat, CH content, and combined water content increase. Moreover, compared with OPC specimens, BPC specimens show lower AS, electrical resistivity, hydration heat, CH, and combined water content and similar late-age strength and UPV. BPC was suitable for producing SUHPP.

## Figures and Tables

**Table 1:** The chemical composition of the materials.

Oxides	Ordinary Portland Cement (%)	Belite-Rich Portland Cement (%)	Silica Fume (%)	Limestone Filler (%)	Blast-Furnace Slag (%)
CaO	64.7	63.27	0.57	59.19	38.29
SiO_2_	21.1	26.65	94.52	–	36.12
Al_2_O_3_	5.07	2.36	0.73	0.21	14.82
Fe_2_O_3_	3.14	2.85	0.22	–	0.47
MgO	0.89	0.96	0.49	0.45	6.49
Na_2_O	0.19	–	–	–	0.06
TiO_2_	0.22	–	–	–	0.62
SO_3_	1.61	2.12	0.26	–	1.61
Loss onignition	2.32	0.89	1.59	39.21	1.16

**Table 2:** Mineralogical composition of the clinker.

Mineralogical Composition	OPC (%)	BPC (%)
3CaO·SiO_2_ (C_3_S)	59.89	29.06
2CaO·SiO_2_ (C_2_S)	15.31	54.48
3CaO·Al_2_O_3_ (C_3_A)	8.12	1.43
4CaO·AlO_3_·Fe_2_O_3_ (C_4_AF)	9.56	8.67

**Table 3:** Mixture design of sustainable ultra-high-performance paste (SUHPP).

Number	Binders (%)				SAP (%)	Water (%)	Additional Water (%)	SP (%)
	Cement (OPC/BPC)	Silica Fume	Limestone	Slag				
OPC–0SAP	50	10	20	20	0	20	0	1.2
BPC–0SAP	50	10	20	20	0	20	0	1.2
OPC–0.25SAP	50	10	20	20	0.25	20	2.5	1.2
OPC–0.5SAP	50	10	20	20	0.5	20	5	1.2

**Table 4:** Experimental methods and test ranges.

NO.	Experimental Method	Total Number of Test Samples	Standard Deviation	Sample Size	Test Time
1	Compressive strength	36	Within ±0.05% of the indicated load	50 × 50 × 50 mm	3, 7, and 28 days
2	Autogenous shrinkage coupled with relative humidity and temperature	4	0.001 μm/m; ±0.5% RH; ±0.1 °C	Ø29 × 430 mm	1–7 days
3	Isothermal calorimetry	4	±20 μW	5 g paste	72 h
4	X-ray diffraction	4	λ = 1.5406 Å 2θ = 0.013°	Powder	28 days
5	Thermogravimetric analysis	4	0.1 μg	Powder	28 days
6	Attenuated total reflectance–Fourier-transform infrared spectroscopy	4	±0.01 cm^−1^	Powder	28 days
7	Ultrasonic pulse velocity	36	0.5 μs	50 × 50 × 50 mm	1, 3, 7, and 28 days
8	Electrical resistivity	12	±0.2 to ±2 kΩ cm	Ø100 × 200 mm	1, 3, 7, and 28 days

**Table 5:** The mass of calcium hydroxide (CH) and combined water (W).

Samples	CH (g/g)	Combined Water (g/g)
OPC–0SAP	3.44%	7.90%
BPC–0SAP	2.99%	7.62%
OPC–0.25SAP	3.95%	8.38%
OPC–0.5SAP	4.32%	8.94%

## Data Availability

The data presented in this study are available from the corresponding author upon a reasonable request.
